# Microbial Level and Microbiota Change of Laver in Dried Laver Processing Line During Production Seasons

**DOI:** 10.3390/foods14030399

**Published:** 2025-01-26

**Authors:** Yi Ding, Feifei Zhou, Renjie Zhou, Qiqi Wang, Saikun Pan, Wenbin Wang

**Affiliations:** 1 Jiangsu Key Laboratory of Marine Bioresources and Environment, Jiangsu Ocean University, Lianyungang 222005, China; 2 Jiangsu Marine Resources Development Technology Innovation Center, Lianyungang 222042, China; 3 Co-Innovation Center of Jiangsu Marine Bio-Industry Technology, Jiangsu Ocean University, Lianyungang 222005, China; 4 Jiangsu Key Laboratory of Marine Biotechnology, Jiangsu Ocean University, Lianyungang 222005, China

**Keywords:** *Porphyra yezoensis*, high microbial load, nutrient, food contact surfaces, cross-contamination, 16S rRNA sequencing

## Abstract

To understand better the high microbial load in dried laver (*Porphyra yezoensis* or nori), this study analyzed the aerobic plate count (APC), coliform count, temperature change, and microbiota of processing water, laver materials, and food contact surface (FCS) samples from three processing plants during the dried laver processing season from December 2023 to April 2024. The seawater used for the first washing had a low microbial load (APCs < 1–2.85 log CFU/g; coliform < 1 log CFU/g) and was dominated by Proteobacteria, Firmicutes, and Bacteroidota. The microbial load of fresh laver (4.21–4.76 log CFU/g) remained unchanged after seawater washing, but significantly increased after continuous shredding, sponge dehydration, first drying, and with the seasonal temperature rise. The microbiota of laver before drying was vulnerable between processing steps and seasons, but consistently shifted back to fresh laver microflora and was dominated by Flavobacteriaceae after drying. The FCSs (except for the curtain), which had a high microbial load (APCs 5.25–8.26 log CFU/g; coliform 1.52–4.84 log CFU/g) with similar microbiota to seawater, caused the secondary contamination of laver during processing. This study revealed the microbial proliferation of laver and seawater microflora in the continuous processing line with high nutrients and with the seasonal processing water temperature rise caused by the local weather, highlighting the need for routine cleaning and sanitizing, better washing of fresh laver, and low temperature control for future dried laver production.

## 1. Introduction

*Porphyra yezoensis* is a large, nutritious, edible seaweed species, rich in protein, vitamin B, and taurine [1]. It is an important ingredient of foods such as seasoned and roasted seaweed, sushi, and kimba. These foods are widely popular in Asian countries and are becoming popular in the global market. The increasing acceptance of seaweed foods in Western countries is mainly attributed to their high nutritional and health value, low carbon footprint, and sustainability [2]. At present, China, Japan, and South Korea mainly breed and process laver products. In recent years, the high microbial load and food safety problems of seaweed foods have drawn increasing attention [3,4,5]. Microbial analysis and high-throughput sequencing results showed that the high microbial load of roasted seaweed was due to the fact that the raw dried laver had high aerobic plate counts (APCs) [6]. A high microbial load of dried laver was also observed in South Korea [7,8]. Our previous study of a laver processing plant in the spring season showed that the APC of laver significantly increased in the drying process and that the APC of dried laver reached 6.90 ± 0.87 log CFU/g [9].

Post-drying decontamination technologies of dried laver have shown limited decontamination under the premise of not sacrificing quality [9,10,11]. However, effective microbial control measures are not available during dried laver production due to the limited understanding of the formation of high microbial load in the dried laver [7,8]. The production of dried laver involves harvesting fresh laver from the sea, removing impurities, washing with seawater, dehydration, shredding and washing with tap water, dehydration, temporary storage, blending with tap water, molding into pieces, sponge dehydration, first hot air drying, second hot air drying, packing, and storage. The production seasons lasts from December to April due to the seasonality of *P. yezoensis* cultivation in the open sea [12]. A Korean research team analyzed laver materials during processing and discovered that the APC and coliform count of laver increased as the processing progressed, and they speculated contamination by sponges due to their insufficient replacement [13]. They also observed that the APC and coliform count of laver increased after 4 days of continuous processing, suggesting that microbial contaminants primarily result from cross-contamination during processing [14].

Although the limited previous studies proposed some possible microbial contamination sources in Korean dried laver processing, the seasonal microbial load and microbiota change in dried laver during the whole production season in China is not fully understood. The impact of seasonal temperature changes, processing water hygiene, and processing line hygiene, particularly the contamination of food contact surfaces, have not been comprehensively evaluated. In addition, the results of previous microbial analysis were based on culture-dependent methods, while the microbial composition of these samples for safety concerns was unknown. Over the past decade, high-throughput sequencing has become a powerful tool for studying microbial sources in food production. 16S rRNA gene amplicon sequencing revealed changes in microbial population and community structure during bacon processing [15]. Another study in a meat processing plant analyzed the inter-relationship of microbiota present in meat, personnel, machinery, and other slaughtering environment samples, and uncovered the sources and transmission routes of microbial populations during meat processing. For example, *Moraxella* spp. was most likely transferred from food contact surfaces (FCSs) such as the gloves of the employees [16].

Therefore, to reveal the formation of high microbial load and the microbiota of dried laver during production, the processing water, harvested fresh laver, laver under processing, dried laver products, and environmental samples of three dried laver processing plants were collected throughout the processing season. The microbial load (APC and coliform count) of these samples was analyzed by culture-based plating methods. Furthermore, the weather and processing water temperature were recorded. Finally, the microbial diversity of these samples from one processing plant was further studied by 16S rRNA sequencing.

## 2. Materials and Methods

### 2.1. Study Design

**Figure 1:**
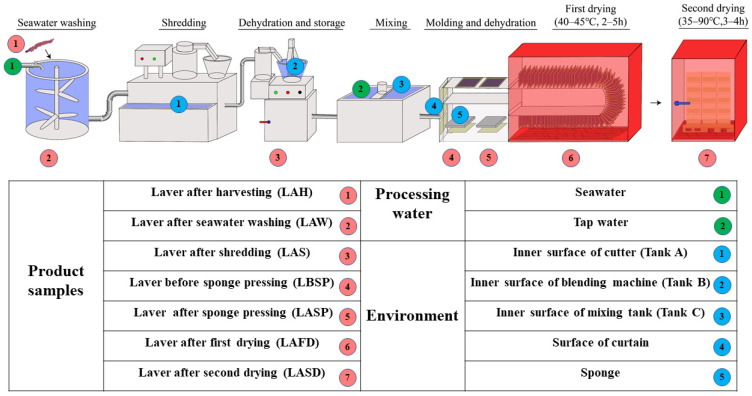
Production workflow and sampling points of dried laver processing plants. The water samples were collected from the hose in the processing workshop with sterile containers. Environment samples (except for the sponges) were collected with swabs.

Three dried laver processing plants with large-scale production located in Lianyungang, Jiangsu, China, participated in this study. Plant A was located on Gaogong Island, Lianyun District, Lianyungang, while Plants B and C were located in the Zhewang Laver Industrial Park, Ganyu District, Lianyungang. The two sampling sites represent the two main dried laver production bases (Lianyun District and Ganyu District) and also two production models (out of and in the industrial park) in Lianyungang, which is one of the main dried laver production bases in China. The three plants produce dried laver in the same way. The workers harvest seaweed from different regions of the offshore seaweed breeding base every day, immediately transport the seaweed to the plant, and manually select large debris from the seaweed. Then, the seaweed is washed in the precipitated seawater to remove the sediment and sand, and processed as shown in Figure 1. The production line does not stop unless the fresh seaweed is insufficient. The material samples for this study included processing water (seawater and tap water) and laver material (after seawater washing, shredding, first drying, and second drying) (Figure 1). The environmental samples were collected from the FCSs including the inner surface of the cutter, the blending machine, and the mixing tank, the surface of the curtain sheet, and the sponge.

### 2.2. Sample Collection

Processing water and laver material samples were collected from the three plants during the production season from December 2023 to April 2024, with three visits per month per plant. FCS samples were collected in March and April 2024. During each visit, three samples with two replicates (*n* = 6) were collected from each sampling point. Seawater (5 L) and tap water (5 mL) were collected from the hose in the processing workshop with sterile containers. Laver material and sponge samples were picked up with sterile gloves and transferred to sterile stomacher bags. FCSs (5 cm × 5 cm) were sampled by premoistened sterile cotton swabs (Huankai Microbial, Guangzhou, China), with 10 horizontal and 10 vertical strokes. All samples were placed in an ice bag immediately after collection and sent to the microbiology laboratory within 4 h. Seawater and tap water samples (5 mL each) were taken for the APC and coliform examination. The remaining seawater was filtered with a sterile filter membrane (0.22 μm). The filter membranes were replaced according to the flow during filtration, and the filter membranes for the same sample were collected in a 15 mL sterile centrifuge tube. One duplicate of the collected sample was used for the APC and coliform analysis on the same day of sampling and another was stored at −80 °C before 16S rRNA sequencing.

### 2.3. Culture-Dependent Microbial Analysis

The APC and coliform count were tested as follows.. Briefly, 25 g of each material or water sample was weighed and transferred to a sterile stomacher bag containing 225 mL sterile saline in a biosafety cabinet (1300 Series A2 Class II; Thermo Scientific, Waltham, MA, USA). The mixture was homogenized in a smasher (HX-4GM; Huxi Industrial Co., Ltd., Shanghai, China) for 2 min. For the swab samples, the tube was thoroughly oscillated. A series of 10-fold dilutions was prepared with sterile saline, and 1 mL of each dilution was evenly mixed with Plate Count Agar (Huankai Microbial, Guangzhou, China) and Violet Red Bile Salt Agar (VRBA, Huankai Microbial, Guangzhou, China). After solidification, double layers of the VRBA plates were formed by adding a further 5 mL culture medium to prevent colony spread. The APC and VRBA plates were turned over and cultured at 36 ± 1 °C for 48 and 24 h, respectively. After culture, 10 colonies on VRBA were selected, confirmed by inoculating in Brilliant Green Lactose Bile Salt Broth (Huankai Microbial, Guangzhou, China), and cultured at 36 ± 1 °C for 24–48 h.

### 2.4. 16S rRNA Sequencing and Bioinformatics Analysis

Samples collected from dried laver processing Plant A were further analyzed by 16S rRNA sequencing as they had a higher microbial load in raw materials, semi-finished products, and finished products. The HiPure Soil DNA kit (Magen, Guangzhou, China) was used to extract total DNA from the food and environmental samples. The amplification of the rRNA gene 16S rRNA V3–V4 region (~466 bp) with primers 341F (CCTACGGGNGGCWGCAG) and 806R (GGACTACHVGGGTATCTAAT) [17] was conducted according to the standard protocol of Gene Denovo Biotechnology Co., Ltd. (Guangzhou, China). The purified amplicon was pooled on Illumina Novaseq 6000 for paired-end sequencing (PE250). All sequencing data were uploaded to the NCBI database (PRJNA1183975). The bioinformatics data analysis was conducted as described in our previous study [6]. Specifically, raw data were filtered using FASTP [18] (version 0.18.0). The paired-end clean reads were merged using FLASH [19] (version 1.2.11). Clean tags were clustered into operational taxonomic units (OTUs) of ≥97% similarity using UPARSE [20] (version 9.2.64). The chimeric tags were removed using the UCHIME algorithm [21], and the taxonomy was annotated by the SILVA database (version 132). The host contamination of chloroplast and mitochondria genes from the laver was excluded [22].

### 2.5. Statistical Analysis

The microbiological test results of each sampling point obtained from three visits per month were averaged. The microbiological test results were converted to log 10 CFU/g or log 10 CFU/cm^2^ and expressed as mean ± standard deviation. For laver materials before drying, according to the yield after two-step drying in the factory, the microbial load was uniformly calculated based on the dry weight of laver (multiplied 15×). For the swab samples, the results were calculated based on the average number of colonies divided by the dilution times and the wipe area (25 cm^2^). In 16S rRNA sequencing, three parallel samples were analyzed. The variance analysis of microbial load among samples was performed by SPSS version 26 (IBM, Armonk, NY, USA), with significance taken at *p* < 0.05. For α-diversity analysis, differences among multiple groups were tested using the Kruskal–Wallis method, whereas differences between two groups and multiple groups were tested using Welch’s *t* test and Tukey’s HSD test, respectively. For β-diversity analysis, Adonis (permutational analysis of variance) was used to test the variance among multiple groups, whereas Welch’s *t* test was used for differences between two groups.

## 3. Results

### 3.1. Microbiological Analysis of Processing Water, Materials, and FCS Samples

**Figure 2:**
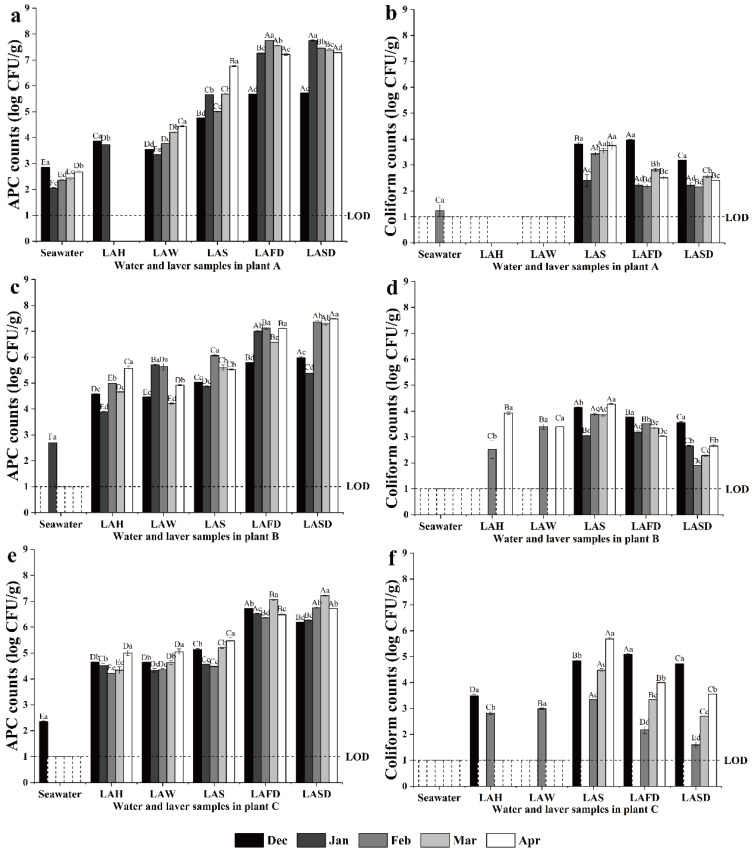
Changes in APC and coliform counts of samples collected in plant A (**a**,**b**), B (**c**,**d**), and C (**e**,**f**) from December 2023 to April 2024. Different capital letters on each column represent significant differences between different processes in the same month, and different lowercase letters represent significant differences between different months for the same process. LAH, laver after harvesting; LAW, laver after seawater washing; LAS, laver after shredding; LAFD, laver after first drying; LASD, laver after second drying.

The microbial load of laver material in Plant A significantly increased (*p* < 0.05) as production and season progressed (Figure 2a,b). The APC of seawater ranged from 2.06 to 2.85 log CFU/g, while that of tap water was <1 log CFU/g. The APC of laver after seawater washing (LAW) was comparable to that of laver after harvesting (LAH) (3.35–4.44 log CFU/g); it increased by 1–2 log CFU/g for laver after shredding (LAS); it also increased by 2–3 log CFU/g for laver after first drying (LAFD); but it was unchanged for laver after second drying (LASD) (Figure 2a). Coliform counts were mostly <1 log CFU/g in seawater (except February), tap water, LAH, and LAW, but increased to the highest level in LAS (2.41–3.75 log CFU/g) and gradually decreased during the two-step drying (Figure 2b). The APC of laver in processing increased from winter to spring. In December, the APC of LAW, LAS, LAFD, and LASD was 3.54, 4.76, 5.69, and 5.72 log CFU/g, and increased by 1.0, 2.0, 1.52, and 1.56 log CFU/g in April, respectively (Figure 2a). Specifically, the APC of seawater and LAW decreased from December to January, and gradually increased from January to April. Similar seasonal trends were observed for the coliform count of LAS, LAFD, and LASD (Figure 2b).

The microbial level of laver material in Plant B significantly increased (*p* < 0.05) as production and seasons progressed (Figure 2c,d). The APC and coliform count of seawater (except January) and tap water were <1 log CFU/g. The APC of LAW was comparable to LAH (4.21–5.63 log CFU/g). It increased by 1–2 log CFU/g for LAS and further increased 2–3 log CFU/g for LAFD, but was unchanged for LASD (Figure 2c). Coliforms in LAH and LAW were not observed except in February and April. The highest coliform counts were found during whole seasons for LAS (3.05–4.27 Log CFU/g) and subsequently decreased by 1–2 log CFU/g with two-step drying (Figure 2d). The APC of LAH, LAW, LAS, LAFD, and LASD in December was 4.58, 4.47, 5.03, 5.79, and 5.97 log CFU/g, and increased by 0.99, 0.45, 0.49, 1.32, and 1.56 log CFU/g in April, respectively (Figure 2c). The highest and lowest coliform counts of LAS, LAFD, and LASD were, respectively, found in December (4.14, 3.77, and 3.56 log CFU/g) and January (3.05, 3.19, 2.66 Log CFU/g) (Figure 2d).

The microbial level of laver material in Plant C significantly increased (*p* < 0.05) as production and seasons progressed (Figure 2e,f). The APC and coliform count of seawater (except APC in December) and tap water were <1 log CFU/g. The APC of LAW was comparable to LAH (3.86–5.05 log CFU/g); however, it increased by 0.5–1 log CFU/g for LAS and by 1–2 log CFU/g for LAFD, but was unchanged for LASD (Figure 2e). Coliform counts of LAH and LAW were not detected except in December and February (2.82–3.49 log CFU/g). The coliform counts increased to the highest level throughout the processing seasons for LAS, but subsequently decreased with two-step drying (Figure 2f). The APC of laver before drying decreased from December to January or February, but increased in March and April (Figure 2e). Coliform counts of LAS, LAFD, and LASD were detected throughout the processing seasons. Specifically, the coliform count was highest (expect for LAS) in December (4.84, 5.09, and 4.74 log CFU/g, respectively), after which it decreased to <1 log CFU/g for each in January, and gradually increased from February to April (5.68, 4.0, and 3.55 log CFU/g, respectively) (Figure 2f).

**Figure 3:**
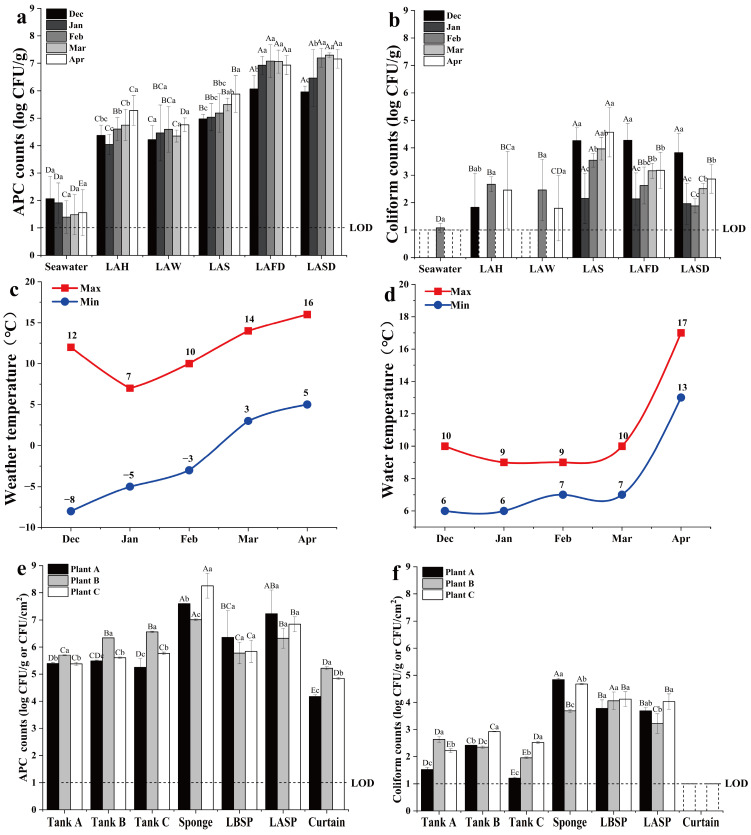
Changes in the APC (**a**) and coliform counts (**b**) of material samples, weather temperature (**c**), and the water temperature of the mixing tank (**d**) in the three plants throughout the processing seasons. Refer to the caption of Figure 2 for an explanation of the sample labels and abbreviations. Changes in the APC (**e**) and coliform counts (**f**) for FCS samples of the three laver processing plants in March and April. Tank A, inner surface of the cutter; Tank B, inner surface of the blending machine; Tank C, inner surface of the mixing tank; LBSP, laver before sponge pressing; LASP, laver after sponge pressing; Curtain, surface of the curtain sheet.

The average microbial load of the three plants significantly increased (*p* < 0.05) as production and seasons progressed (Figure 3a,b). Low APC was detected in seawater but coliforms were seldom detected. The APC and coliform count were < 1 log CFU/g in tap water. The APC of laver before shredding (4.21–4.76 log CFU/g) did not significantly increase until the shredding step (4.97–5.87 Log CFU/g), and further increased after the first drying (6.06–7.29 log CFU/g) (Figure 3a). Coliforms were occasionally detected at a low level in laver before shredding, but were often detected in the post-shredding steps. Specifically, the coliform count increased to the highest level after shredding (4.57 log CFU/g), and then gradually decreased with the two-step drying (Figure 3b).

From December to April, the APC of laver samples before drying gradually increased, but after drying, there was no further increase after February (Figure 3a). For laver samples after shredding and the two-step drying, coliform count was highest (expect for LAS) in December, decreased to the lowest level in January, and gradually increased from February to April (Figure 3b). Similar trends were observed with the local weather temperature and the water temperature of the mixing tank, which decreased from December to January and then steadily increased from January to April (Figure 3c,d).

The FCS samples from the three plants collected in spring (March–April) showed that the APC of the sponges was highest (up to 8.26 log CFU/g), and the APC of Tanks A–C ranged from 5.25 to 6.56 log CFU/cm^2^, which was significantly higher (*p* < 0.05) than the APC of the curtain (4.17–5.22 log CFU/cm^2^) (Figure 3e). Coliforms were detected on the inner surface of Tanks A–C (1.52–2.92 log CFU/cm^2^) in the three plants, except for the curtain (Figure 3f). Just after the laver was dehydrated using sponges, the APC increased by 1 log CFU/g (Figure 3e), while the coliform count remained stable. Considering the small weight loss of laver after sponge dehydration, and the fact that the sponges had 10–100 higher APC but similar coliform count than the laver before sponge pressing, it was clear that the laver was contaminated by the sponges in all the three plants.

### 3.2. Microbiome Analysis of Processing Water, Laver Materials, and FCS Samples

**Figure 4:**
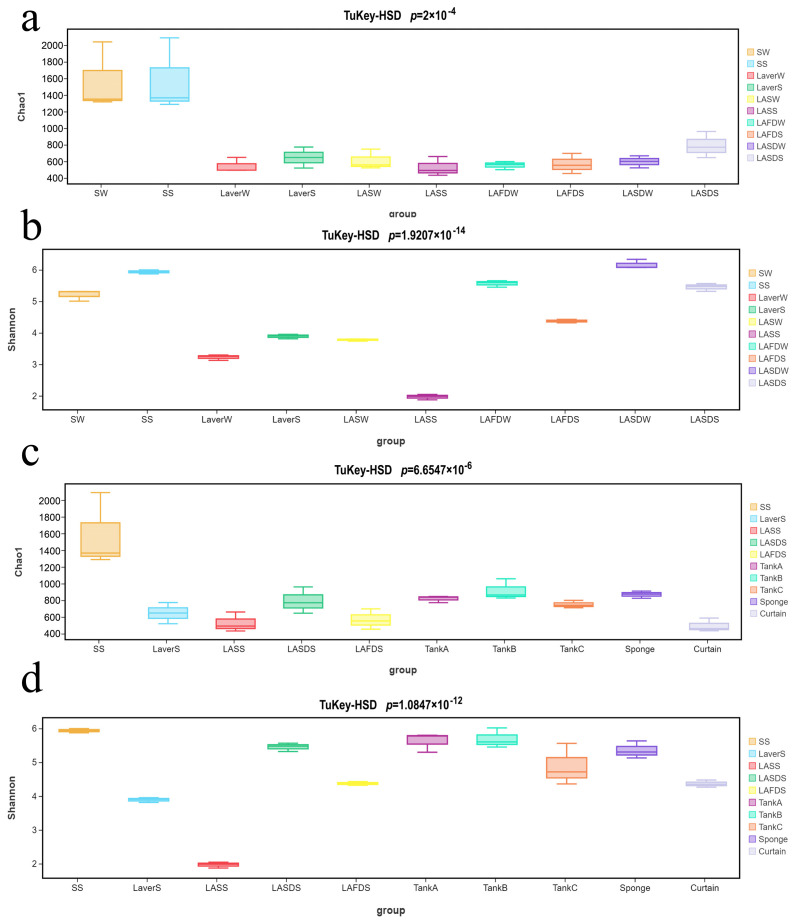
The α-diversity (Chao1 and Shannon indices) of microbiota present in raw materials (**a**,**b**) and environmental samples (**c**,**d**) collected from Plant A. W and S in the last letter, respectively, represents sample collected in winter and in spring. SW, seawater in winter; LaverW, the laver after seawater washing in winter. Refer to the captions of Figure 2 and Figure 3 for an explanation of the other sample labels and abbreviations.

Samples collected from laver processing Plant A were further analyzed by 16S rRNA sequencing, because of their higher microbial loads. The effective reads per sample ranged from 105 878 to 121 626. The Chao1 and Shannon indices were, respectively, used to quantify the microbial species richness and the richness and evenness of the collected samples. Tukey’s HSD test (for multiple groups) and Welch’s *t* test (for two groups) based on the Chao1 indices showed that the microbial richness of seawater (SS and SW) was significantly higher than for the other material samples (*p* < 0.05). The microbial richness of material samples and FCS samples (except for the curtain) collected in the spring was comparable (*p* > 0.05), and was higher than that of the curtain (Figure 4a,c).

Tukey’s HSD test and Welch’s *t* test based on the Shannon indices showed that the microbial species richness and evenness of seawater and laver after drying were significantly higher than those in the other laver samples in the same season. The microbial species richness and evenness of material and FCS samples also showed significant differences (*p* < 0.05). Specifically, the microbial species richness and evenness of seawater, LASDS, and FCS samples (except curtain) were the highest (*p* > 0.05), while those of LASS was the lowest (Figure 4b,d).

**Figure 5:**
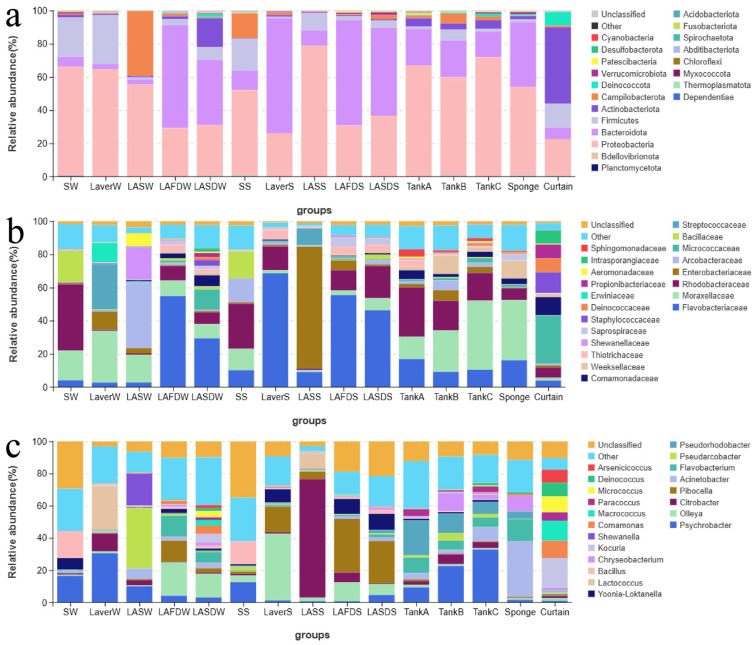

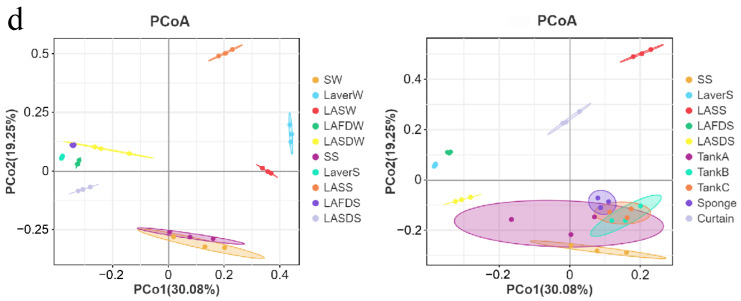
The microbial composition at the phylum (**a**), family (**b**), and genus (**c**) level and principal co-ordinate analysis (PCoA) (**d**) of the raw materials and environmental samples collected from Plant A. Refer to the captions of Figure 2, Figure 3 and Figure 4 for an explanation of the sample labels and abbreviations.

Figure 5 showed the microbial composition and similarity of microbiota (PCoA) of the collected samples in Plant A. The microbiota of seawater, material, and environmental samples (except for the curtain) were dominated by Proteobacteria, Bacteroidota, and Firmicutes. The microbiota of the curtain samples was dominated by Actinobacteriota, followed by Proteobacteria, Firmicutes, Deinococcus-Thermus, and Bacteroidota (Figure 5a). Seawater in winter (SW) and spring (SS) clustered together in PCoA analysis and showed similar microbiota (Figure 5d), which was dominated by Rhodobacteraceae (Proteobacteria, 27.11–39.82%), such as *Yoonia*–*Loktanella* (0.88–6.92%); Moraxellaceae (Proteobacteria, 12.83–18.03%), such as *Psychrobacter* (12.65–16.62%); Bacillaceae (Firmicutes, 16.23–18.50%), such as *Bacillus* (13.81–16.33%); and Flavobacteriaceae (Bacteroidota, 4.14–10.23%), such as *Olleya* (0.70–4.09%) (Figure 5a–c).

The microbiota of laver after seawater washing (Laver) and after shredding (LAS) varied between processing steps and seasons, as reflected by the scattered plot in PCoA analysis (Figure 5d). The dominant bacteria of laver after seawater washing in winter (LaverW) were Moraxellaceae (31.06%), such as *Psychrobacter*; Enterobacteriaceae (Proteobacteria, 10.96%), such as *Citrobacter*; and Streptococcaceae (Firmicutes, 27.34%), such as *Lactococcus*. In contrast, the microbiota of laver after seawater washing in spring (LaverS) was dominated by Flavobacteriaceae (68.61%), such as *Olleya* (41.36%) and *Pibocella* (15.89%). The dominant bacteria of LASW were Arcobacteraceae (Campilobacterota, 39.22%), such as *Pseudarcobacter* (37.33%), and Moraxellaceae (16.75%), such as *Psychrobacter* (10.05%) and *Acinetobacter* (6.36%). The microbiota of LASS was dominated by Enterobacteriaceae (73.37%), such as *Citrobacter* (73.30%) (Figure 5a–c).

The microbiota of LAFD and LASD were consistent between processing steps and seasons, and were also consistent with the microbiota of fresh laver in spring according to the PCoA analysis (Figure 5d). The dominant bacteria were Flavobacteriaceae (29.36–55.44%), such as *Olleya* (6.72–20.75%), *Pibocella* (2.59–33.34%), and *Flavobacterium* (0.86–13.14%); Moraxellaceae (2.88–9.32%), such as *Psychrobacter* (0.8–4.63%) and *Acinetobacter* (1.54–3.55%); Rhodobacteraceae (7.16–19.36%), such as *Yoonia*–*Loktanella* (1.07–10.01%); and Micrococcaceae (Actinobacteriota, 0.43–12.34%), such as *Kocuria* (0.08–5.38%) and *Micrococcus* (0.12–3.86%) (Figure 5a–c).

The PCoA analysis showed that the microbiota of FCS samples including Tanks A–C and sponges were similar with seawater (Figure 5d). The dominant bacteria were Moraxellaceae (13.39–41.67%), such as *Acinetobacter* (2.21–34.7%) and *Psychrobacter* (1.57–32.83%); Rhodobacteraceae (7.1–29.76%), such as *Pseudorhodobacter* (4.46–21.53%); Enterobacteriaceae (0.33–6.22%), such as *Citrobacter* (0.21–6.17%); Flavobacteriaceae (9.2–16.94%), such as *Flavobacterium* (5.73–13.45%); and Weeksellaceae (Bacteroidota, 2.13–11.26%), such as *Chryseobacterium* (0.59–11.24%). The curtain samples showed different microbiota from the other samples. The dominant bacteria were Micrococcaceae (29.03%), such as *Kocuria* (18.15%) and *Micrococcus* (10.05%); Staphylococcaceae (Firmicutes, 12.42%), such as *Macrococcus* (12.4%); Intrasporangiaceae (Actinobacteriota, 8.07%), such as *Arsenicicoccus* (8.05%); Deinococcaceae (Deinococcota, 8.34%), such as *Deinococcus* (8.34%); and Comamonadaceae (Proteobacteria, 10.85%), such as *Comamonas* (10.55%) (Figure 5a–c).

## 4. Discussion

The results of the culture-based method used in this study showed that the background microbial level of laver ranged from 4.21 to 4.76 log CFU/g (based on dry weight) after the seaweed was harvested from the sea and did not significantly change after the seawater washing process. This indicated that the current washing by constant agitation removed the sediment but not the bacteria on the laver, and so better washing methods for the decontamination of microbes deserve further study [23]. However, the microbial load of laver steadily increased after shredding and temporary storage, sponge dehydration, and the first drying (6–8 log CFU/g) throughout the processing season. The increased APC of dried laver along with the processing steps was consistent with previous studies [9,24]. The high coliform count of laver was mainly detected after shredding and before the two-step drying process, which was consistent with the high abundance of Enterobacteriaceae in laver after shredding detected by the 16S rRNA sequencing. The increase in microbial load in laver after the shredding step was due to the release of nutrients such as protein and saccharides from the shredded laver in the tap water with low salinity [25], considering the low APC of seawater and tap water. The mold and yeast count of laver samples in this study were low (1.40–1.88 log CFU/g) and are not displayed.

A seasonal change in the microbial level of laver materials was observed in this study. From December to April, the APC of laver before drying gradually increased. The coliform count of laver samples after shredding decreased from December to January and then steadily increased from January to April, which was consistent with the local weather temperature and the water temperature trend in the plant. This suggests that temperature promotes the proliferation of microbes in the shredded laver materials, which is well understood in other food processing plants such as for meat and aquatic products [26]. In these plants, special processing temperature and time control are essential to inhibit both the microbial growth of initial microorganisms and the formation of toxins such as staphylococcal enterotoxins and histamine [27]. Although the water temperature in laver processing is comparable with that of meat (11–15 °C in the boning room) or aquatic product (12 °C) processing workshops [28], a temperature rise will also promote microbial proliferation in FCSs due to a lack of routine cleaning.

The microbiota of laver after seawater washing, laver after shredding, and laver after temporary storage varied between processing steps and seasons, indicating that the microbiota of laver was probably affected by many unknown factors. However, the microbiota of laver after the first and second drying was consistent between processing steps and seasons. The microbial composition is accordant with our previous finding that the microbiota of dried laver and finished roasted seaweed products were dominated by Flavobacteriaceae, Moraxellaceae, and Rhodobacteraceae [6]. Furthermore, the microbiota of laver after drying was consistent with the microbiota of fresh laver (after seawater washing) in spring, indicating that the dried laver microbiota shift back during the first drying process, which could be due to the high nutritional content in shredded laver, the better utilization of these nutrients by native microflora [29], high water activity, suitable temperature (40–50 °C), and time (2.5 h). It is encouraging that no well-known pathogen was found in the laver materials during processing, but the contamination with coliforms before the drying process should be controlled because bacteria like *Salmonella* can survive the drying process [30]. Based on the results of this study, *Acinetobacter* (3.55–2.39%) and *Citrobacter* (0.64–0.61%), which were abundant in the laver after shredding, survived the second drying. Our previous study also showed that *Acinetobacter* survived the roasting process of seaweed sandwiches [6].

A cross-contamination of laver materials from FCSs during processing was observed in this study. Firstly, in all sampled FCSs in this study (except for the curtain), high APC (5.25–8.26 log CFU/g) and coliform levels (1.52–4.84 log CFU/g) were detected. These levels were higher than the accepted microbial criteria for FCSs after routine cleaning and sanitizing, although the criteria varied among studies. The APC ranged from 1 CFU/cm^2^ for direct contact with prepared food to 10^3^ CFU/cm^2^ for cutting boards and knives in restaurants [31]. The coliform count ranged from 0 per slide to 1 log CFU/cm^2^ [32]. Furthermore, the APC of laver increased (Ca. 1 log CFU/g) after sponge pressing. The repeated use of sponges and production lines lead to the accumulation of nutrients and bacteria, which lead to the contamination of processing lines and cause secondary contamination to laver.

The microbiota of FCS samples (except for the curtain) was similar to seawater, except that it was dominated by Moraxellaceae such as *Acinetobacter* and *Psychrobacter.* This indicated that the microflora of seawater, especially moraxellaceae, are more competitive to proliferate and colonize the laver processing line with high moisture and nutrients [28]. Similarly to the results of laver in processing, no well-known pathogen was found in the laver materials during processing except for the sponges on which a high abundance of *Acinetobacter* was detected. *Acinetobacter* is widely distributed in the environment, including food processing facilities [33], especially in humid environments [34]. This opportunistic bacterium is a causal agent of hospital-borne infections and is resistant to antimicrobial substances, desiccation and disinfection regimes, and host immune responses [35].

The contamination of laver from FCSs with a high microbial load and coliform bacteria *Citrobacter* and opportunistic bacterium *Acinetobacter* highlighted the inadequate routine cleaning and sanitizing in dried laver processing plants. Routine cleaning and sanitizing procedures are critical in preventing the cross-contamination of foods by pathogens and food poisoning [36]. This is because an appropriate cleaning practice could remove large and small debris, dirt and microorganisms, and possible food and shelter for pests [27]. However, routine cleaning was largely missing during the current dried laver production. Sponge replacement in the three plants ranged from up to 7 days in winter to 1–2 days in spring, which is aimed to avoid the sponge being too sticky to peel off the laver after drying. It has been reported that the APC of dried laver dramatically increased after 4 days of continuous processing in the plants [14], which indicated the close relationship of high microbial load with the contamination of the processing line. The primary reason for the lack of routine cleaning and sanitizing procedures in dried laver production is the fact that the formation of high microbial load in dried laver is not well understood; for example, understanding is limited to its increase in the drying step [37]. Another reason is that dried laver belongs to a group of primary agricultural products that do not have limits for APC and coliform count in China and South Korea [7]. In addition, processing is urgent due to the harvesting of a large quantity of fresh laver in the limited production seasons. The implementation of improved cleaning protocols relies on the establishment of good manufacturing practices and sanitation standard operating procedures through the collaboration of the government, the seaweed industry, and the research institutions.

## 5. Conclusions

With culture-dependent and culture-independent microbial analysis, this study showed that background microorganisms of laver and seawater proliferated in the production line with the release of laver nutrients after the shredding process. The continuous processing without adequate routine cleaning and sanitizing and the seasonal process water temperature rise caused by the local weather further promoted the microbial proliferation and secondary contamination of the laver. This process led to the high microbial load and distinctive microbial community of dried laver products and food contact surfaces. The microbiota of laver in processing was variable before drying but consistently shifted back to Flavobacteriaceae after the first drying. The microbiota of food contact surfaces (except for the curtain) was similar to the microbiota of seawater. These results highlight the need of future research on the adoption of routine cleaning and sanitizing, more efficient washing of fresh laver, and temperature control in future dried laver processing.

## Data Availability

All sequencing data were uploaded to the NCBI database (PRJNA1183975). The other raw data supporting the conclusions of this article will be made available by the authors on request.
